# Moderate Drought Stress Induces Increased Foliar Dimethylsulphoniopropionate (DMSP) Concentration and Isoprene Emission in Two Contrasting Ecotypes of *Arundo donax*

**DOI:** 10.3389/fpls.2017.01016

**Published:** 2017-06-13

**Authors:** Matthew Haworth, Stefano Catola, Giovanni Marino, Cecilia Brunetti, Marco Michelozzi, Ezio Riggi, Giovanni Avola, Salvatore L. Cosentino, Francesco Loreto, Mauro Centritto

**Affiliations:** ^1^Tree and Timber Institute, National Research CouncilSesto Fiorentino, Italy; ^2^Department of Agrifood Production and Environmental Sciences, University of FlorenceSesto Fiorentino, Italy; ^3^Institute of Biosciences and Bioresources, National Research CouncilSesto Fiorentino, Italy; ^4^Dipartimento di Agricoltura, Alimentazione e Ambiente, Università degli Studi di CataniaCatania, Italy; ^5^Department of Biology, Agriculture and Food Sciences, National Research CouncilRome, Italy

**Keywords:** photosynthesis, stomatal conductance, methionine pathway, chlorophyll fluorescence, dimethylsulphide, giant reed, biomass crop

## Abstract

The function of dimethylsulphoniopropionate (DMSP) in plants is unclear. It has been proposed as an antioxidant, osmolyte and overflow for excess energy under stress conditions. The formation of DMSP is part of the methionine (MET) pathway that is involved in plant stress responses. We used a new analytical approach to accurately quantify the changes in DMSP concentration that occurred in two ecotypes of the biomass crop *Arundo donax* subject to moderate drought stress under field conditions. The ecotypes of *A. donax* were from a hot semi-arid habitat in Morocco and a warm-humid environment in Central Italy. The Moroccan ecotype showed more pronounced reductions in photosynthesis, stomatal conductance and photochemical electron transport than the Italian ecotype. An increase in isoprene emission occurred in both ecotypes alongside enhanced foliar concentrations of DMSP, indicative of a protective function of these two metabolites in the amelioration of the deleterious effects of excess energy and oxidative stress. This is consistent with the modification of carbon within the methyl-erythritol and MET pathways responsible for increased synthesis of isoprene and DMSP under moderate drought. The results of this study indicate that DMSP is an important adaptive component of the stress response regulated via the MET pathway in *A. donax*. DMSP is likely a multifunctional molecule playing a number of roles in the response of *A. donax* to reduced water availability.

## Introduction

Dimethylsulphoniopropionate (DMSP) is an important part of sulfur metabolism in photosynthetic organisms (Giovanelli, 1987; Stefels, 2000) and the precursor of the volatile organic compound (VOC) dimethyl sulphide (DMS) (Bentley and Chasteen, 2004). Approximately half of the global flux of sulfur to the atmosphere occurs in the form of DMS (Malin et al., 1992), and DMS plays a key role in the climate system by acting as a condensation nucleus (Keller et al., 2012). Despite the central role played by DMSP in the global sulfur cycle, comparatively little is known about its role in plant growth or potential dynamics in response to plant stress. Previous analyses of DMSP have focused on marine phytoplankton and plants where it is found in higher concentrations. This has led to a hypothesis that DMSP formation competes with the emission of isoprene during stress (Dani and Loreto, 2017). If replicated in vascular plants, this would have major implications for our understanding of the regulation VOC emissions during stress. However, analysis of DMSP in higher plants, particularly under drought stress, has been neglected, and as a consequence the role of DMSP is unclear.

**FIGURE 1:**
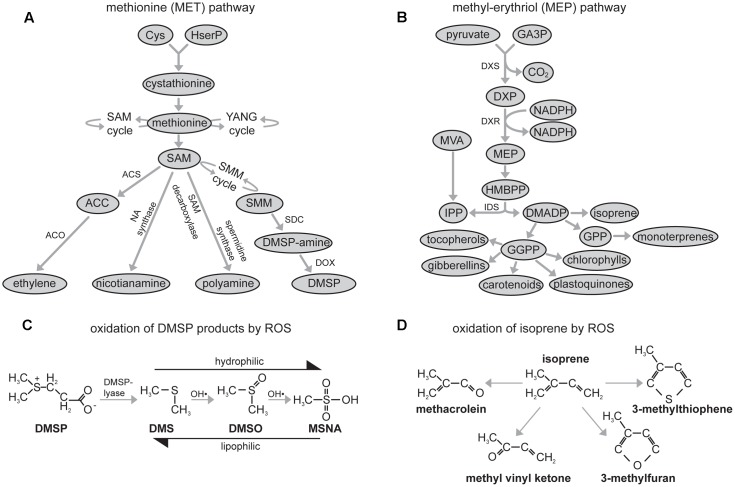
Schematic diagrams of: **(A)** the methionine (MET) pathway (DMSP, dimethylsulphoniopropionate; SAM, *S*-adenosylmethionine; SMM, *S*-methylmethionine; NA, nicotianamine; PA, polyamine; ACS, Aminocyclopropane-1-carboxylic acid synthase (ACC synthase); ACO, ACC oxidase; SDC, SMM decarboxylase; DOX, DMSP-amine oxidase; HserP, O-phosphohomoserine), the stages involving synthesis of SMM and DMSP-amine occur outside the chloroplast envelope before DMSP-amine is transported into the chloroplast envelope where DMSP is formed (Amir et al., 2002); **(B)** oxidation of DMSP products by ROS (DMS, dimethylsulphide; DMSO, dimethylsuphoxide; MSNA, methane sulphinic acid) (Stefels, 2000; Sunda et al., 2002; Bentley and Chasteen, 2004); **(C)** the methyl-erythritol (MEP) pathway (GA3P, glyceraldehyde 3-phosphate; DMADP, dimethylallyl diphosphate; DXR, 1-deoxy-D-xylulose 5-phosphate reductoisomerase; GPP, geranyl diphosphate; GGPP, geranylgeranyl diphosphate; DXP, 1-deoxy-D-xylulose 5-phosphate; DXS, 1-deoxyxylulose 5-phosphate synthase; HMBPP, (*E*)-4-hydroxy-3-methylbutyl-2-enyl pyrophosphate; MVA, mevalonate synthesized via the mevalonate pathway contributing to IPP) (Lichtenthaler et al., 1997), all stages other than MVA and the formation of gibberellins occur within the chloroplast, and; **(D)** oxidation of isoprene by ROS (Jardine et al., 2012).

The function of DMSP is not well defined (Stefels, 2000), but it may serve as a metabolically compatible osmolyte (Kirst, 1996; Van Bergeijk et al., 2003), an antioxidant (Sunda et al., 2002; Husband and Kiene, 2007; Husband et al., 2012), a defensive compound against herbivores (Van Alstyne and Houser, 2003), a sink for excess sulfur (Stefels, 2000), a methyl donor in transmethylation reactions (Challenger et al., 1957; Giovanelli, 1987) or an overflow for excess energy (Groene, 1995; Stefels, 2000). DMSP is part of the methionine (MET) pathway (**Figure 1A**; James et al., 1995; Trossat et al., 1996). The MET pathway is an important component in the regulation of plant metabolism linked to the production of the antioxidant glutathione (Leustek and Saito, 1999), regulation of iron metabolism via the action of the amino acid nicotianamine (Curie et al., 2009; Klatte et al., 2009), the influence of polyamine on cellular signaling of abiotic stress (Alcázar et al., 2010) and the production of the stress hormone ethylene (Sato and Theologis, 1989). Given the importance of the MET pathway to metabolic adaptation to environmental stress, improved analysis of the dynamics of DMSP would contribute to our understanding of this critical component of plant metabolism.

During drought events the availability of soil water declines and plants close stomatal pores to reduce transpirative water-loss. The decrease in stomatal conductance (*G*_s_) reduces the availability of CO_2_ at the active site of carboxylation in the chloroplast envelope and lowers rates of photosynthesis (*P*_N_) (Centritto et al., 2011b; Lauteri et al., 2014; Dbara et al., 2016; Killi et al., 2017). Plant stress during drought is often the result of excess of energy as the amount of intercepted radiation utilized in photochemistry declines, and increasing amounts of energy instead induce oxygen photoreduction forming dangerous reactive oxygen species (ROS) (Pinheiro and Chaves, 2011; Zivcak et al., 2013). During the initial stages of drought, plants may utilize the emission of isoprene derived from the plastidic 2-*C*-methyl-D-erythritol 4-phosphate pathway (MEP) (**Figure 1B**) to neutralize ROS (**Figure 1C**). Isoprene can strengthen thylakoid membranes (Velikova et al., 2011) and act directly as an antioxidant (**Figure 1D**) quenching biochemical reactions with ROS and reactive nitrogen species (Vickers et al., 2009a). The carotenoid xanthophylls are also part of the MEP pathway and are involved in the dissipation of excess energy via non-photochemical quenching (NPQ) (Cousins et al., 2002). DMSP may also exert an antioxidant function (Sunda et al., 2002; Husband and Kiene, 2007; Husband et al., 2012). *Solanum lycopersicum* leaves subject to severe drought exhibited a 75% increase in foliar concentrations of DMSP (Catola et al., 2016), consistent with antioxidant (Sunda et al., 2002; Husband and Kiene, 2007) or overflow (Stefels, 2000) functions for DMSP. Dimethlysulphide has been proposed to act as a delocalised antioxidant, stabilizing the lipid phase of photosynthetic and cellular membranes during oxidative stress (Catola et al., 2016) in a manner similar to isoprene (Velikova and Loreto, 2005). The subsequent oxidation products of DMS by ROS, dimethylsuphoxide (DMSO) and methane sulphinic acid (MSNA), are increasingly hydrophilic. Therefore, the degradation products of DMSP have differential partitioning between lipid and aqueous phases, and may consequently protect different cellular compartments from oxidative stress (Sunda et al., 2002). Acrylate, a by-product of DMSP cleavage by DMSP-lyase (**Figure 1B**), is also a precursor for ethylene, and increased concentrations of acrylate stimulate ethylene release (Plettner et al., 2005). During drought emissions of ethylene generally decline (Habben et al., 2014), possibly indicating reduced availability of DMSP due to oxidation by ROS (**Figure 1B**).

A new analytical technique developed by Catola et al. (2016) now allows rapid and accurate analysis of low concentrations of DMSP. We utilized this approach to investigate the response of DMSP in the giant reed (*Arundo donax*) subject to moderate drought stress in field conditions. *Arundo donax* is a fast growing isoprene emitting (Haworth et al., 2017b) member of the Poaceae family that shows potential as a biomass crop (Mantineo et al., 2009). The rapid biomass accumulation of *A. donax* is sustained by high rates of CO_2_ assimilation (Haworth et al., 2017b). However, these levels of *P*_N_ are accompanied by high rates of stomatal conductance (*G*_s_) and transpirative water-loss that require a high level of water availability (Haworth et al., 2017b). In comparison to other biomass crops such as *Populus* (e.g., Kauter et al., 2003; Centritto et al., 2011a), little is known regarding the physiological and metabolic response of *A. donax* to drought, and the potential for varietal differences in this response that may be exploited in the commercial development of *A. donax* as a biomass crop. Information regarding the dynamics of DMSP may allow the modification of the MET pathway in *A. donax* to optimize the physiological response to drought stress and maximize growth. We selected two *A. donax* ecotypes from a temperate and a more arid habitat (Sesto Fiorentino, Central Italy, and Marrakesh, Morocco, respectively) that had shown contrasting responses to severe drought in a previous study (Haworth et al., 2017a). These *A. donax* ecotypes were planted in a common garden field trial and subjected to moderate drought to: (i) investigate the impact on leaf gas exchange characteristics; (ii) quantify the effect on photochemical and non-photochemical energy usage using chlorophyll fluorescence; (iii) examine any change in the emission of the VOC isoprene, (iv) study the response of DMSP to drought, and; (v) propose a hypothesis regarding the possible role of DMSP in the drought response of *A. donax* and the potential to utilize this response, and any varietal differences found in this study, to optimize biomass production of *A. donax* in drought prone semi-arid marginal lands. We hypothesize that the MET pathway plays a key role in the drought response of *A. donax* via increased production of DMSP (e.g., Catola et al., 2016).

## Materials and Methods

### Field Site and Experimental Design

Rhizomes were collected from clonal populations from a warm sub-Mediterranean humid region, Sesto Fiorentino, Florence, in Central Italy, that receives 800 mm of precipitation each year and has a mean summer (June to August) temperature of 23°C, and from an arid pre-desert area of Marrakesh, Morocco, that receives 200 mm of precipitation each year and has a mean summer temperature of 30°C. The same ecotypes were exposed to severe drought in another study and exhibited differences in xylem morphology associated with the response to soil drying (Haworth et al., 2017a). The rhizomes were cut into equal portions of 20 cm in length (at least one bud was visible on each portion) and planted in a common garden experiment at the experimental farm of the University of Catania, Sicily (37° 25′N 15° 03′E), in March 2015. The rhizomes were planted at a depth of 15 cm every 0.5 m in rows spaced 0.8 m apart in 4.0 m × 3.0 m sized plots. Six plots of each of the Italian and Moroccan *A. donax* ecotypes were planted (12 plots in total), and to avoid edge effects a 2.4 m border of three rows of *A. donax* was placed around the field. The field was unfertilised and all plants were irrigated equally to field capacity until July 2015 when the rhizomes had established and the stems were roughly 1.5 m in height. On the 7th July irrigation was ceased to half of the field; three plots each of the Italian and Moroccan ecotypes were rain-fed, and the remaining three plots for each ecotype continued to receive irrigation. Supplementary irrigation was equivalent to 100% of potential evapotranspiration (ETc) during July to August, calculated each day as:

$$\text{ETc}={\text{E}}_{\text{o}}*{\text{K}}_{\text{p}}*{\text{K}}_{\text{c}}$$

Where E_o_ is the evaporation of water from a class-A pan (mm); K_p_ is the pan coefficient, and; K_c_, the crop growth stage (between 0.4 and 1.9) (Triana et al., 2015). Daily rain-fall was subtracted from the daily calculation of water to be supplied as irrigation (Allen et al., 1998). Soil samples were collected on the same day as the leaf samples were collected from 0 to 90 cm depth and the soil water content determined gravimetrically (Klute, 1986; Killi et al., 2014). Full details of the site and soil conditions at the experimental farm are given in Cosentino et al. (2006).

### Leaf Gas Exchange and Chlorophyll Fluorescence

Six weeks after the cessation of supplementary irrigation to half of the *A. donax* plants, analyses of leaf gas exchange and chlorophyll fluorescence parameters were performed. Measurements of *P*_N_, *G*_s_, the sub-stomatal concentration of [CO_2_] (*C*_i_) and electron transport rate (ETR) were performed on the mid-section of the second newest fully expanded leaf using a LiCor Li6400XT fitted with a 2 cm^2^ leaf cuvette (Li-Cor, Inc., Lincoln, NE, United States). Two replicate plants from the centre of three plots were analyzed for each ecotype and treatment (*n* = 6). Environmental parameters were controlled and the following settings were used: 400 ppm [CO_2_], 2000 μmol m^-2^ s^-1^ of photosynthetically active radiation (PAR, 10% blue and 90% red light), and leaf temperature of 30°C. To reduce diffusive leaks through the chamber gasket, a supplementary gasket was added and the IRGA exhaust air was fed into the interspace between the chamber and the supplementary gaskets. The ETR was calculated as:

ETR=ΦPSII×PPFD×α×β

where: PPFD is the photosynthetic photon flux density (μmol m^-2^ s^-1^); ΦPSII is the actual quantum yield of photosystem II; α is the leaf absorbance (a standard value of 0.85 was used), and; β is the partitioning of electrons between photosystem I and II (assumed to be 0.5) (Genty et al., 1989). The respiration in the dark (*R*_N_) was estimated 10 min after switching off the light unit in the cuvette, when CO_2_ emission from the leaf had stabilized (e.g., Shi et al., 2015). Gas-exchange measurements were performed between 10.00 and 12.00 each day, when the plants exhibited the highest levels of *P*_N_ and *G*_s_. The maximum (*F*_v_/*F*_m_) and the actual quantum yield of photosystem II (ΦPSII: ΔF/F’m), and the dissipation of light energy as NPQ were recorded using a Hansatech FMS-2 (Hansatech, King’s Lynn, United Kingdom) after 30 min of dark adaptation (Genty et al., 1989).

### Isoprene Emission

The emission of isoprene was measured in the field from the same leaves of *A. donax* used for gas-exchange analysis, under the same environmental settings, but a LiCor Li6400 fitted with a 6 cm^2^ cuvette and LED light unit was used. When monitoring isoprene emission, air from the cuvette with the enclosed leaf passed through a biphasic adsorbent trap containing 30 mg of Tenax and 20 mg of Carboxen (GERSTEL GmbH & Co.KG, Germany). A pump (Elite 5, A.P. Buck, Orlando, FL, United States) was used to pass 2 L of air through each trap at a rate of 200 ml min^-1^. Measurements of the concentration of isoprene in the ambient air (blanks) were performed using an empty leaf cuvette before and after each measurement. The traps were then stored at 4°C prior to analysis in the laboratory. Isoprene was first desorbed from traps at high temperature and then measured using a gas chromatograph – mass spectrometer (GC-MS) with an Agilent HP-INNOWAX (30 m × 0.32 mm × 0.15 μm) GC column. A 5977A mass selective detector with electron ionization operating at 70eV was used for analysis. Isoprene was identified by matching the spectrum peak with a library spectral database (NIST 11.L) and through comparison of the retention time and mass spectrum with an isoprene analytical standard (Sigma Aldrich, St. Louis, MO, United States) injected into the GC-MS at different concentrations. The isoprene analytical standard was also used to construct a calibration curve by injecting known concentrations of isoprene into the GC-MS. The data was analyzed using Agilent MassHunter Workstation software (Agilent 7890A, Agilent Technologies, Santa Clara, CA, United States). The concentration of isoprene within the leaf was calculated using the approach of Singsaas et al. (1997).

### Analysis of Dimethylsulphoniopropionate (DMSP) and Relative Water Content (RWC)

Leaf samples were collected after completion of the leaf gas exchange and chlorophyll fluorescence measurements. The first fully most expanded leaf was collected from the same two plants in each plot (*n* = 6 for each ecotype and treatment) adjacent to the leaf that had been used for physiological analysis. The lower 4–5 cm of each leaf was removed to be used for determination of foliar relative water content (RWC) following the protocol of Diaz-Pérez et al. (1995). The remainder of the leaf was flash frozen in liquid nitrogen before being stored at -80°C prior to analysis of DMSP by solid phase micro extraction from head-space (HS-SPME). An in-depth description of the method is provided by Catola et al. (2016). Briefly, the leaf samples were ground in liquid nitrogen. An aliquot (0.2 g) of each sample was then placed inside a 20 ml screw-cap head-space vial (Agilent Technologies, Santa Clara, CA, United States), together with 250 μl 0.5 M NaOH, 2 g of NaCl and sufficient distilled water to make up a total 5 ml volume. Teflon coated silicon septa (Agilent Technologies) were used to seal the head-space vials, which were then incubated at 60°C for 12 h, to allow complete hydrolysis of DMSP to DMS. A three-phase divinylbenzene/carboxen/polydimethylsiloxane (Supelco, Bellafonte, PA, United States) 75 μm width, 2 cm long, solid phase micro-extraction fiber was placed in the head-space of the vials for 10 min at 40°C. To ensure consistent sampling and mixing of DMS in the head-space of the vials, a Gerstel MPS2 XL auto-sampler (Gerstel GmbH & Co. KG, Mülheim an der Ruhr, Germany) was used. The VOCs adsorbed by the fiber were analyzed using an Agilent 7820 GC-chromatograph with a 5977A M5D with electron ionization running at 70 eV and a HP-Innowax column (50 m, 0.2 mm, ID 0.4 μm DF). Dimethyl sulfide was identified via comparison with a spectral database library (NIST11.L) and injection of a known standard of DMS into the GC-MS (Sigma Aldrich). An example chromatograph of DMS is given in the Supplementary Data. A calibration curve was constructed by injecting increasing concentrations of DMS into the GC-MS and the amount of DMS used to infer the concentration of DMSP within the leaves.

### Statistical Analysis

Statistical analyses were performed using SPSS 20 (IBM, Armonk, New York, NY, United States). To test the effect of water deficit on the Moroccan and Italian *A. donax* ecotypes we used a two-way ANOVA (Supplementary Information) and a one-way ANOVA with an LSD *post hoc* test to assess differences in variance between samples associated with either ecotype or treatment effects.

## Results

**FIGURE 2:**
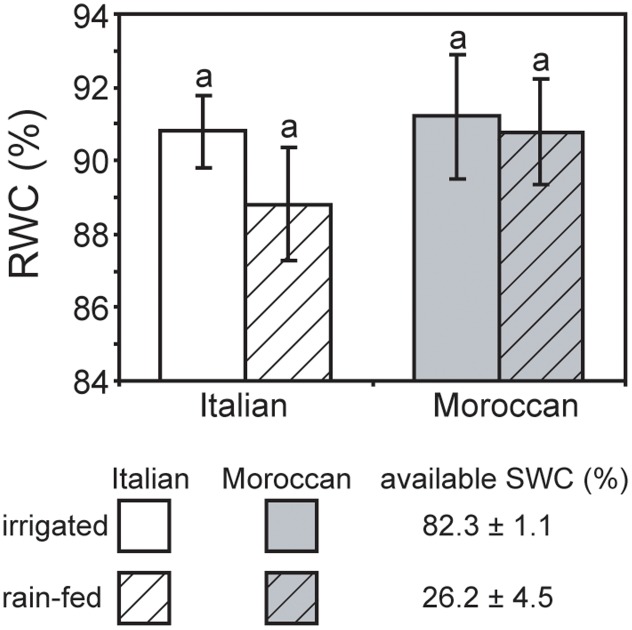
Relative water content of leaves of Moroccan (gray) and Italian (white) *Arundo donax* ecotypes grown in the field under irrigated control (open) and rain-fed drought (hatched) conditions. Error bars indicate one standard error either side of the mean (*n* = 6). Letters indicate homogenous groups determined using a one-way ANOVA and LSD *post hoc* test.

**FIGURE 3:**
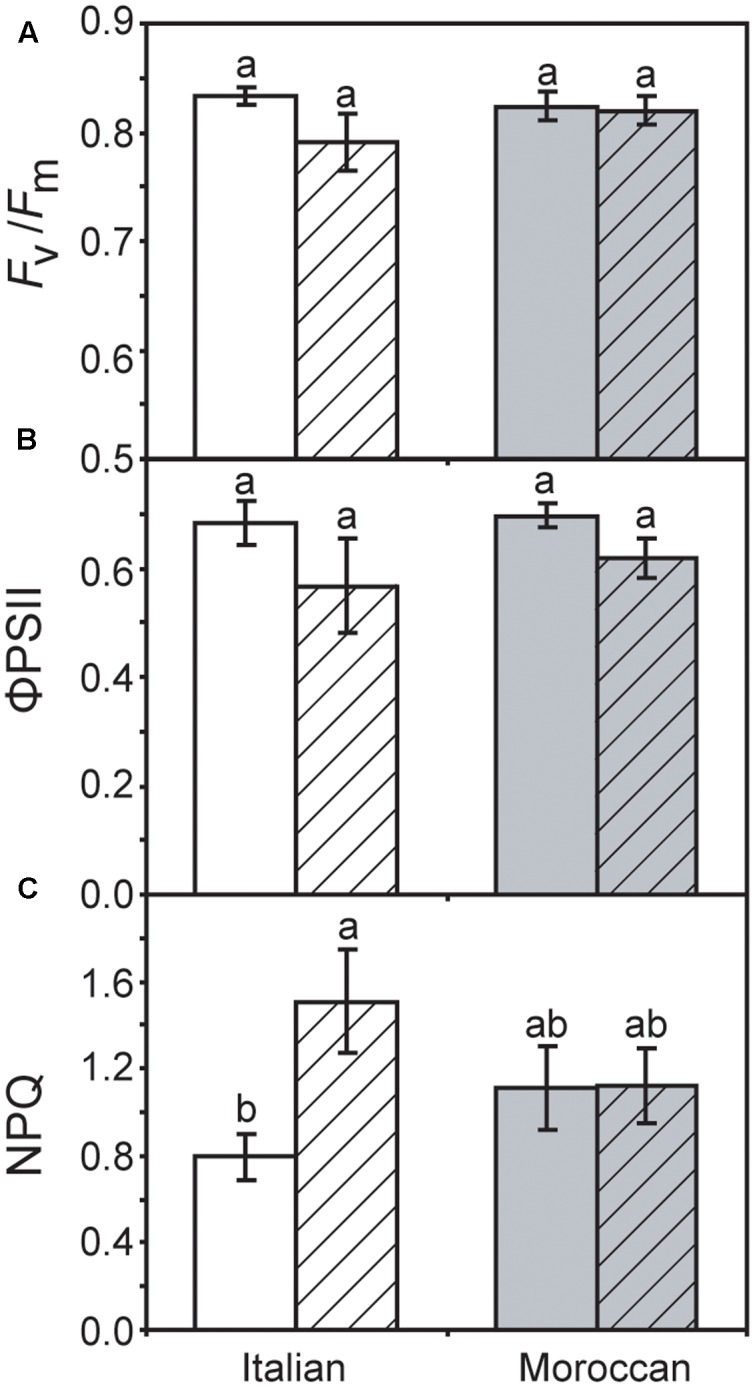
Chlorophyll fluorescence measurements of Moroccan (gray) and Italian (white) *A. donax* ecotypes grown in the field under irrigated control (open) and rain-fed drought (hatched) conditions: **(A)** maximum quantum efficiency of PSII (*F*_v_/*F*_m_); **(B)** actual quantum efficiency of PSII (ΦPSII), and; **(C)** non-photochemical quenching (NPQ). Error bars indicate one standard error either side of the mean (*n* = 6). Letters indicate homogenous groups determined using a one-way ANOVA and LSD *post hoc* test.

**FIGURE 4:**
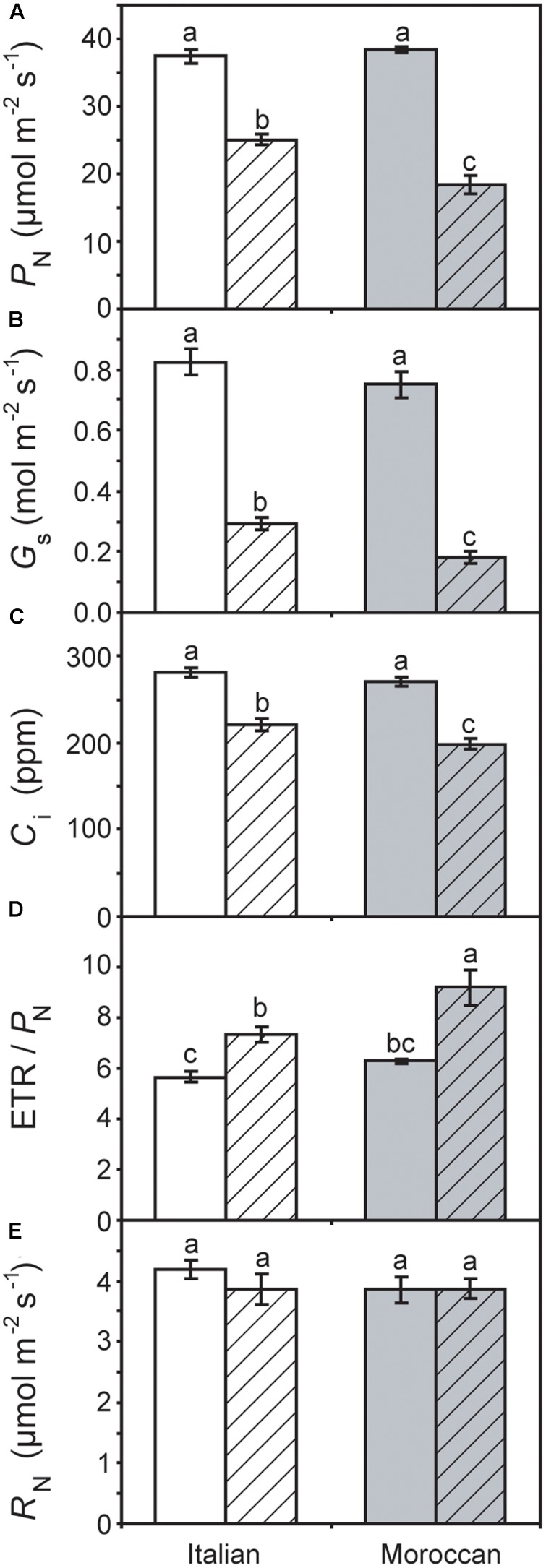
Leaf gas exchange and simultaneous chlorophyll fluorescence parameters of Moroccan (gray) and Italian (white) *A. donax* ecotypes grown in the field under irrigated control (open) and rain-fed drought (hatched) conditions: **(A)** photosynthesis (*P*_N_); **(B)** stomatal conductance (*G*_s_); **(C)** internal sub-stomatal concentration of CO_2_ (*C*_i_); **(D)** the ratio of electron transport rate (ETR) to *P*_N_, and; **(E)** respiration in the dark (*R*_N_). Error bars indicate one standard error either side of the mean (*n* = 6). Letters indicate homogenous groups determined using a one-way ANOVA and LSD *post hoc* test.

Growth under rain-fed field conditions resulted in a reduced RWC of *A. donax* leaves (particularly in the Italian ecotype), but the difference in comparison with leaves of irrigated plots was not statistically significant (**Figure 2**). The maximum (*F*_v_/*F*_m_) (**Figure 3A**) and actual (ΦPSII) (**Figure 3B**) quantum yields of PSII were also not significantly affected by the cessation of supplementary irrigation, although the two parameters were more reduced again in the Italian than in the Moroccan ecotype. Under rain-fed conditions, the Italian ecotype exhibited a significant 90.0% increase in the dissipation of energy as heat (as shown by the parameter NPQ), while the Moroccan ecotype showed no response (**Figure 3C**). Photosynthesis (**Figure 4A**) and *G*_s_ (**Figure 4B**) showed no difference between the *A. donax* ecotypes under irrigated conditions. However, under moderate drought stress the Moroccan ecotype exhibited more pronounced reductions in *P*_N_ and *G*_s_ (52.0 and 75.5%), in comparison to the Italian ecotype (33.0 and 64.5%). Reductions in *G*_s_ resulted in lower *C*_i_ in both *A. donax* ecotypes, especially in the Moroccan (**Figure 4C**). The drought stressed Moroccan ecotype also exhibited an ETR/*P*_N_ ratio higher than the Italian. However, the ETR/*P*_N_ ratio was not different in the ecotypes under irrigated conditions. No ecotype or treatment effect was observed in *R*_N_ (**Figure 4D**).

**FIGURE 5:**
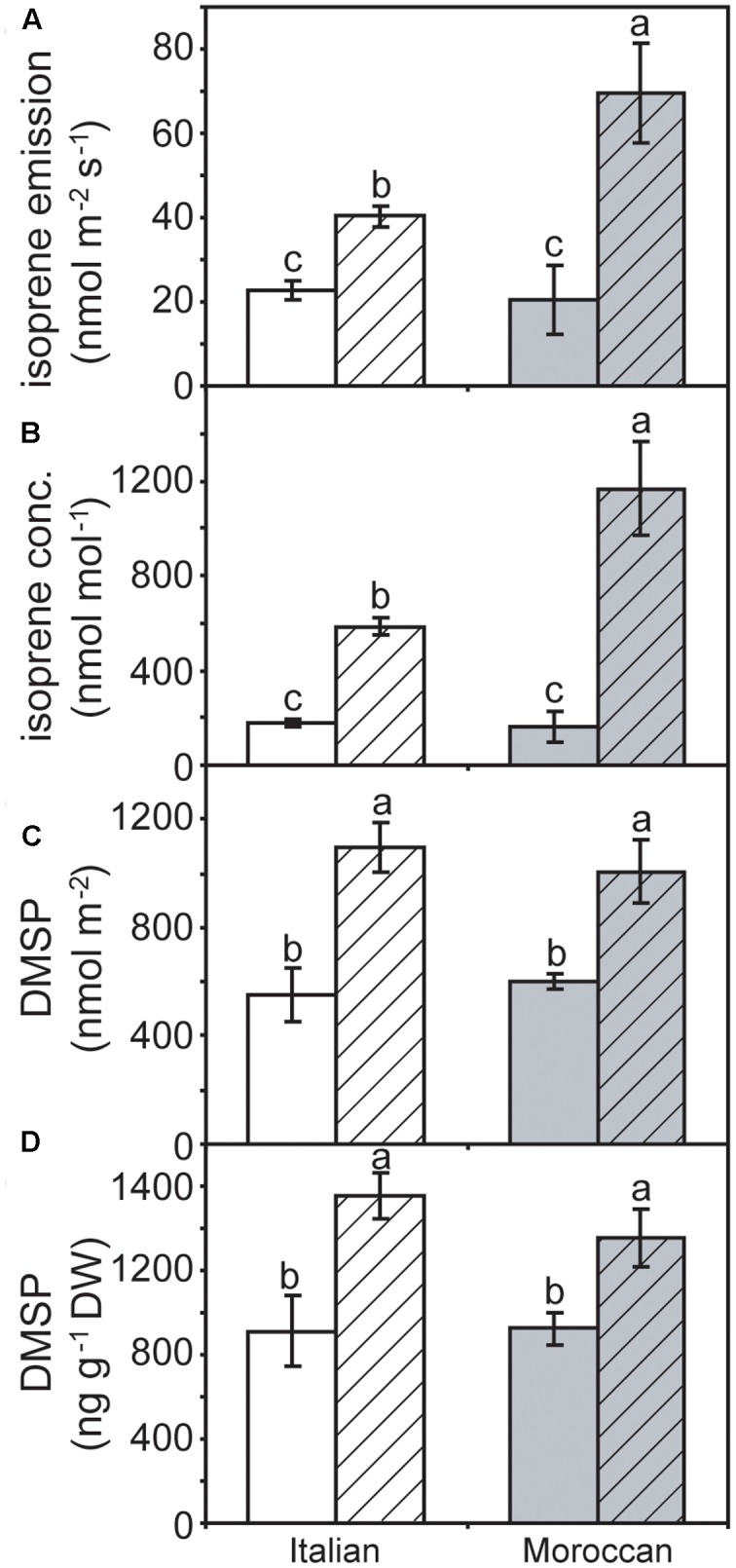
**(A)** Isoprene emission, **(B)** the concentration of isoprene within the sub-stomatal air-spaces inside the leaf, **(C)** dimethylsulphoniopropionate (DMSP) as a function of leaf area, and **(D)** concentration of DMSP per unit dry mass of leaf matter in Moroccan (gray) and Italian (white) *A. donax* ecotypes grown in the field under irrigated control (open) and rain-fed drought (hatched) conditions. Error bars indicate one standard error either side of the mean (*n* = 6). Letters indicate homogenous groups determined using a one-way ANOVA and LSD *post hoc* test.

No difference was observed in the rate of isoprene emission between the two *A. donax* ecotypes under irrigated conditions. However, the emission of isoprene was significantly enhanced under rain-fed conditions, by 236.4% in the Moroccan ecotype, and to a lesser extent (76.4%) in the Italian ecotype (**Figure 5A**). A similar pattern was also observed in the concentration of isoprene within the leaf (**Figure 5B**). The amount of DMSP when determined on a leaf area basis was significantly greater in rain-fed than irrigated plants. This increase was 98.4 and 67.3% in the Italian and Moroccan ecotypes, respectively (**Figure 5C**). A similar increase in the concentration of DMSP was also recorded in both *A. donax* ecotypes under rain-fed conditions, when a dry weight basis was used (**Figure 5D**). No significant difference in the response of DMSP was observed between the two *A. donax* ecotypes, both when irrigated and rain-fed. The results of a two-way ANOVA of gas exchange, chlorophyll fluorescence, DMSP and isoprene parameters are presented in the Supplementary Information.

## Discussion

*Arundo donax* has potential to be a productive biomass crop in many warm to hot regions (Angelini et al., 2009; Mantineo et al., 2009), which are, however, often exposed to recurrent drought. The *A. donax* plants examined in this study showed no effect of drought on foliar RWC, and comparatively minor reductions in leaf gas exchange parameters in comparison to other studies (e.g., Cosentino et al., 2016; Haworth et al., 2017b); therefore the drought stress experienced by the plants in this study was considered to be moderate for *A. donax*. A number of studies have examined the effect of severe drought on *A. donax* (e.g., Sánchez et al., 2015; Haworth et al., 2017b), but comparatively few have investigated the response of this species to moderate drought over a sustained period, which may be more representative of the majority of situations where water availability limits growth in the field (Farooq et al., 2009; Yan et al., 2016). As such, the results of this study provide valuable physiological and biochemical insights into the response of *A. donax* to moderate drought under field conditions.

### Impact of Moderate Drought Stress on Photosynthesis

Previous studies have reported comparatively little ecotypic variation in the leaf gas exchange and chlorophyll fluorescence parameters of contrasting *A. donax* ecotypes when subjected to severe drought (i.e., where *G*_s_ falls below 10% of control values) (Sánchez et al., 2015; Haworth et al., 2017a). In contrast, we have observed statistically significant ecotypic differences, albeit comparatively minor, in *P*_N_, *G*_s_, *C*_i_ and ETR/*P*_N_ responses of the Italian and Moroccan *A. donax* ecotypes to moderate drought (**Figure 4**). Despite both ecotypes showing no significant differences in RWC (**Figure 2**), stem height or stem number per rhizome (data not shown), the Moroccan ecotype generally exhibited more pronounced reductions in leaf gas exchange parameters under the moderate drought stress in the rain-fed treatment. The Moroccan ecotype has been shown to increase xylem vessel size during drought, while the Italian ecotype reduced xylem vessel size (Haworth et al., 2017a). This contrast in the morphological response of the water transport systems could be related to the adaptation of the *A. donax* ecotypes to their respective environments. The increase in xylem vessel size found in the Moroccan *A. donax* may promote the loss of above ground photosynthetic tissues via enhanced xylem embolism (e.g., Cochard, 2002; Cochard et al., 2002) to preserve the viability of the rhizome in an environment where drought is more severe and its onset more rapid. In contrast, the reduction in the xylem vessel diameter in the Italian ecotype would favor increased resistance to xylem embolism and the retention of leaves and stems (Tyree and Sperry, 1989). The lower *G*_s_ observed in the Moroccan ecotype under moderate drought stress (**Figure 4B**) may be due to a higher rate of xylem embolism promoting stomatal closure than in the Italian *A. donax* (Cochard, 2002). The Moroccan ecotype also exhibited a higher ETR/*P*_N_ ratio under drought, indicative of a greater proportion of electrons being utilized in photorespiration (Sun et al., 2014). However, no difference was observed in *F*_v_/*F*_m_ values of both ecotypes under irrigated and rain-fed conditions (**Figure 3A**) and alongside reduced *G*_s_ (**Figure 4B**) suggest that the limitations to *P*_N_ under moderate drought were mostly diffusive (Centritto et al., 2003; Aganchich et al., 2009). However, the Italian ecotype did exhibit increased dissipation of heat as NPQ alongside a lower increase in isoprene emission under rain-fed conditions, in comparison to the Moroccan ecotype. Increased isoprene formation during drought might have helped maintain *P*_N_ limitations only at the diffusion level (limited by stomatal closure and low *C*_i_; **Figure 4**), avoiding the increase of NPQ and the probable resultant photochemical damage. Indeed, isoprene emitters show lower NPQ than non-emitters under physiological and stress conditions, especially under drought (Beckett et al., 2012; Pollastri et al., 2014). This possibly indicates lower oxidative stress at the chloroplast thylakoid level and enhanced protection of photochemistry of photosynthesis (Velikova et al., 2011) associated with high isoprene emission in the Moroccan ecotype. Severe drought stress has been shown to induce an increase in the proportion of photorespiration and *R*_N_ relative to *P*_N_, with absolute values of photorespiration and *R*_N_ declining as drought progresses (Centritto et al., 2011a; Sun et al., 2014; Killi et al., 2017). The moderate drought stress imposed on the *A. donax* did not appear sufficient to induce any alteration in *R*_N_ values (**Figure 4E**).

### Dynamics of Dimethylsulphoniopropionate and Isoprene Concentrations under Moderate Drought Stress

Under severe drought stress in the field the rate of isoprene emission in *A. donax* did not increase (Haworth et al., 2017b). However, in many other experiments including this study, isoprene emission and concentration are stimulated by moderate stress conditions, which uncouple isoprene from photosynthesis, its main source of carbon (**Figure 1A**). Isoprene has been proposed to act as a mobile diffusive antioxidant stabilizing chloroplast membranes under stress conditions (Velikova and Loreto, 2005; Vickers et al., 2009b). As already mentioned, the higher synthesis and emission of isoprene in the drought-stressed Moroccan ecotype, in comparison to the Italian ecotype, might be related to avoidance of photochemical damage in leaves where photosynthesis is impaired by diffusive limitations, or due to enhanced xanthophyll function in the Italian ecotype to promote NPQ. The increased emission of isoprene might be more suitable for ecotypes that frequently endure prolonged drought in their natural habitats. Exploitation of differences in isoprene emission in commercial *A. donax* clones would require consideration of the benefits of enhanced protective capacity balanced against increased losses of assimilated carbon.

The results of this study are consistent with the observations of Catola et al. (2016) of stimulated DMSP formation under drought. This enhanced foliar concentrations of DMSP occurred alongside the increase in isoprene emission in the *A. donax* plants in the rain-fed treatment when compared to irrigated plants (**Figure 5D**). This is not consistent with competition between DMSP and isoprene synthesis within the chloroplast proposed to take place in marine phytoplankton (Dani and Loreto, 2017). A previous study at the same site found no difference in isoprene emission rates between irrigated and rain-fed *A. donax* plants (Haworth et al., 2017b). However, the previously analyzed *A. donax* experienced a longer more severe drought stress, while the *A. donax* in the present study experienced a shorter less severe drought that allowed the maintenance of *P*_N_ (**Figure 4A**) and the likely existence of a pool of labile carbon sufficient to sustain enhanced isoprene emission (Barta and Loreto, 2006). The synthesis of isoprene (Banerjee et al., 2013) and DMSP (James et al., 1995; Trossat et al., 1996) reveal activation of two chloroplastic pathways (MEP and MET, respectively). As largely demonstrated for the MEP pathway (Loreto and Schnitzler, 2010), also the MET pathway possibly plays a protective role in moderate drought stress conditions. The increase in DMSP concentration would be consistent with a role as an overflow for excess energy (Stefels, 2000). The increased NPQ in the Italian *A. donax* (**Figure 3C**) is indicative of a reduction in the usage of energy for photochemistry (Haworth et al., 2017b). DMSP is considered to act more strongly as a protective overflow under conditions of low nitrogen availability, facilitating the redistribution of nitrogen to other amino acids via the MET pathway (Groene and Kirst, 1992). As the field where the *A. donax* rhizomes were planted was unfertilised prior to the field trial, this may have promoted the increase in DMSP levels observed during growth under moderate drought stress, when the energy partitioning to photochemistry became unbalanced (Stefels, 2000). The increase in foliar DMSP concentration found in the *A. donax* plants subject to moderate drought in this study may have also acted as an osmolyte reducing the leaf water potential and allowing the maintenance of RWC (Kirst, 1996; Van Bergeijk et al., 2003). This is consistent with observations in the salt-marsh grass *Spartina alterniflora* where the concentration of DMSP did not adjust in response to an increase in oxidative stress (Husband et al., 2012), indicating that the role of DMSP may not be protective.

The MET pathway leads to the formation of many compounds that have well-defined roles in abiotic tolerance. However, it is unclear whether DMSP in higher plants is a by-product of the synthesis of these other MET derived compounds. However, a protective role for DMSP is consistent with its synthesis within the chloroplast. Greater availability of DMSP may also promote enhanced emission of DMS (Husband and Kiene, 2007) to act as a mobile antioxidant protecting thylakoid membranes in a manner similar to isoprene (Velikova and Loreto, 2005), via the oxidation of DMS to DMSO (Husband and Kiene, 2007). The rate of DMS emission is often correlated to the availability of DMSP (Groene, 1995; Catola et al., 2016); however, the rate of release of DMS is also dependent upon the activity of DMSP-lyase (Stefels et al., 1995) and of bacterial degradation of DMS (Carini, 2016). It is not possible to estimate potential rates of DMS emission based upon the concentrations of DMSP observed in the present study due to the low affinity of DMSP-lyase for DMSP (Stefels et al., 2007; Reisch et al., 2011) Further analytical advances are required to directly determine accurate foliar DMS release at the low levels likely to occur in *A. donax* before more definitive conclusions may be drawn as to the role of DMS and its precursor DMSP as aqueous and gaseous antioxidants.

## Conclusion

*Arundo donax* has typically shown little ecotypic variation in physiological responses to severe drought. However, our analysis of the responses to moderate drought stress under field conditions in *A. donax* ecotypes from warm sub-humid (Central Italy) and hot semi-arid (Morocco) habitats indicated a degree of ecotypic variation, with the Moroccan ecotype exhibiting more pronounced reductions in *P*_N_ and *G*_s_. This may reflect selective pressures experienced by the Moroccan ecotype to preserve the viability of the rhizome in a habitat where droughts develop more rapidly, are more severe and longer in duration. As synthesis of isoprene and DMSP increased significantly under moderate stress, we suggest that the underlying MEP and MET pathways, play an important role in adapting to moderate drought and preserving photosynthetic capacity once the stress is relieved. Modification of MEP and MET pathways may potentially assist in the development of stress resistant and climate-adapted *A. donax* biomass crops.

## Author Contributions

MH, GM, ER, GA, SLC, CB, SC, and MM conducted the experiment. MH, MC, and FL wrote the manuscript.

## Conflict of Interest Statement

The authors declare that the research was conducted in the absence of any commercial or financial relationships that could be construed as a potential conflict of interest.
