# Supplementary material for: Moderate Drought Stress Induces Increased Foliar Dimethylsulphoniopropionate (DMSP) Concentration and Isoprene Emission in Two Contrasting Ecotypes of Arundo donax
Source: Front Plant Sci. 2017 Jun 13;8:1016. doi: 10.3389/fpls.2017.01016 (PMC5468454; doi:10.3389/fpls.2017.01016)
Supplement: Supplementary file 1 [file Data_Sheet_1.DOCX]

Supplementary information figure 1 – a) an example chromatograph showing absorption peaks of dimethylsulphide (DMS); b) DMS absorption calibration curve (each data point represents the mean of three replicates), and; c) an example chromatograph illustrating the comparative difference in the DMS peak area of representative leaf samples of irrigated (black line) and rain-fed (grey line) *Arundo donax*.

Supplementary data – two-way ANOVA of gas exchange, chlorophyll fluorescence, isoprene and DMSP parameters of Italian and Moroccan ecotypes of A. donax grown under rain-fed (drought) and irrigated (well-watered) conditions.

gas exchange

|  | *P*_N_ | | *G*_s_ | | *C*i | | ETR/*P*_N_ | |
| --- | --- | --- | --- | --- | --- | --- | --- | --- |
|  | F_3,23_ | *P* | F_3,23_ | *P* | F_3,23_ | *P* | F_3,23_ | *P* |
| water | 281.4 | 5.5 x 10^-21^ | 257.1 | 3.2 x 10^-20^ | 121.4 | 2.3 x 10^-14^ | 34.3 | 5.1 x 10^-7^ |
| variety | 8.5 | 0.00562 | 7.1 | 0.0106 | 7.7 | 0.00820 | 10.7 | 0.00207 |
| water * variety | 15.7 | 0.000263 | 0.3 | 0.609 | 1.1 | 0.310 | 2.6 | 0.117 |

chlorophyll fluorescence

|  | *F*_v_/*F*_m_ | | ΦPSII | | NPQ | |
| --- | --- | --- | --- | --- | --- | --- |
|  | F_3,23_ | *P* | F_3,23_ | *P* | F_3,23_ | *P* |
| water | 2.4 | 0.133 | 3.8 | 0.0589 | 4.2 | 0.0478 |
| variety | 0.4 | 0.518 | 0.5 | 0.495 | 0.04 | 0.849 |
| water * variety | 1.7 | 0.197 | 0.2 | 0.691 | 3.9 | 0.0566 |

DMSP and isoprene

|  | isoprene emission | | isoprene conc. | | DMSP per unit dry mass | | DMSP per unit leaf area | |
| --- | --- | --- | --- | --- | --- | --- | --- | --- |
|  | F_3,23_ | *P* | F_3,23_ | *P* | F_3,23_ | *P* | F_3,23_ | *P* |
| water | 11.6 | 0.00469 | 21.6 | 0.000453 | 16.1 | 0.00130 | 25.8 | 0.000167 |
| variety | 1.9 | 0.192 | 2.9 | 0.111 | 0.5 | 0.498 | 0.1 | 0.818 |
| water * variety | 2.6 | 0.132 | 3.3 | 0.0921 | 0.6 | 0.455 | 0.6 | 0.465 |
